# Preliminary observations on the effect of Kegel exercise and perihip muscle exercise training and combined training on transversus abdominis thickness in healthy young women

**DOI:** 10.1097/MD.0000000000048388

**Published:** 2026-04-17

**Authors:** Yufei Gao, Lili Yu, Cong Chen, Desheng Li, Miao Ye

**Affiliations:** a School of Rehabilitation of Capital Medical University, Beijing, China; b Department of Physiotherapy, Beijing Boai Hospital, China Rehabilitation Research Center, Beijing, China.

**Keywords:** activation of transversus abdominis, hip muscle group, instant effect, joint training, Kegel exercise

## Abstract

**Background:**

The objective of our study was to compare the immediate effects of Kegel exercise alone and 2 types of hip muscle group exercise training with different degrees of contraction as well as combined training on thickness of the transversus abdominis (TrA), and to explore the optimal training method for its activation.

**Methods:**

Twenty-eight healthy young women were selected as research participants, all of whom underwent Kegel exercises, mild-to-moderate hip adductor muscle training, and mild-to-moderate hip external rotation muscle training. Musculoskeletal ultrasound was used to measure resting TrA and immediate TrA thickness after each training session and to compare the changes in thickness after different training methods and times.

**Results:**

There was a significant difference in the thickness of the TrA after only 1 training session compared to that at rest (*P* < .01), and after 5 training sessions compared to that at rest and after only 1 training session (*P* < .01). Comparing the immediate thickness of the TrA in the 5 different training methods, moderate contraction training of the hip external rotation muscle resulted in higher thickness than mild and moderate contraction of the hip adductor muscle and mild contraction of the hip external rotation muscle did (*P* < .01).

**Conclusion:**

Choosing any of the 5 training methods for 1 session can instantly activate the TrA, and a combination of the 5 training methods is more effective than training using solely one method. Among the different training methods, moderate contraction training of the hip external rotation muscle group had the best immediate activation effect on the TrA.

## 1. Introduction

At present, Kegel exercises are a classic pelvic floor muscle rehabilitation training method, by contracting and relaxing the pubococcygeus, or pelvic floor muscle, during this exercise, the stress of urine incontinence was relieved. It is beneficial to enhance the strength of these muscles to contract and stretch as well as the muscle strength in the urethra,^[1]^ and after years of clinical practice and research, it has been proven that Kegel exercises have a positive effect on the treatment and prevention of pelvic floor dysfunction diseases but require high compliance from postpartum women.^[2]^

There is a connection between functioning of the pelvic floor, transversus abdominis (TrA) and pelvic external muscles. Related studies have shown that the TrA and pelvic floor muscles have a synergistic effect and contraction of the pelvic floor muscles is accompanied by involuntary contractions of the TrA and internal oblique muscles.^[3]^ When actively contracting pelvic floor muscles, the participation of the abdominal muscles is a normal response, and there is a common contraction mechanism between the TrA and pelvic floor muscles.^[4]^ In recent years, clinical studies have found a significant correlation between thickness of the TrA and electromyographic activity of the levator ani muscle.^[5]^ Another study suggests that the TrA and pelvic floor muscles exhibit coordinated movement, and changes in the TrA thickness can be used to indicate changes in the pelvic floor muscle contraction status in all tasks.^[6]^ The strengthening training of hip abductor muscle group indirectly activates core muscle group (such as TrA) by optimizing pelvic stability and lower limb power chain conduction, thereby enhancing overall posture control ability.^[7]^ Fasciological research reports the anatomical connection between the pelvic external muscles and pelvic floor muscles, thereby directing the issue of pelvic floor muscles towards the hip and lower limbs.^[8]^ In short, pelvic floor muscle contractions cannot be completely separated from the pelvic external muscle group contractions.

The TrA is located in the deep layer of the lateral abdominis muscle group, with its posterior end originating from the thoracolumbar fascia and its anterior end migrating to the TrA tendon and terminating at the white line.^[9]^ Related studies have shown that appropriate training and activation of the TrA will help properly utilize the surrounding local core muscles, improving the effectiveness of open- and closed-chain movements.^[10]^

Ultrasonography was used to measure the thicknesses of the TrA. Ultrasound imaging has the advantages of simplicity, safety, noninvasiveness, low cost, and good evaluation reliability.^[11]^ Studies have shown a statistically significant correlation between activation of the TrA and its thickness.^[12]^ McMeeken study has demonstrated a good correlation between EMG activity and thickness change in TrA measured using ultrasound scanning. Measures of thickness change may therefore be used as biofeedback or as a tool to investigate the function of this muscle.^[13]^ Evidence-based medicine shows that using musculoskeletal ultrasound to measure muscle thickness can reflect muscle contraction ability and can be used as a noninvasive muscle function assessment method.^[14]^

The clinical significance of TrA activation is relatively high. Compared with traditional treatments such as taking active drugs and medication prescriptions for adults with chronic nonspecific lower back pain, a specific program based on pre-activation and reeducation exercises for the TrA significantly reduces disability in the short term and improves its activation ability.^[8]^ When the TrA and pelvic floor muscles contract simultaneously, the change in the thickness of the TrA is most significant in patients with chronic low back pain.^[15]^ It may happen because the simultaneous contraction of the TrA and pelvic floor muscles can increase the internal pressure of the waist and abdomen, thereby improving stability of the spine.^[16]^ Lee and Hodges proposed that contraction of the TrA may strengthen the white line, which is a key factor in the rehabilitation of rectus abdominis muscle separation.^[17]^ Related studies have shown that strengthening the TrA can effectively reduce asymmetric movements in stroke patients with hemiplegia.^[18]^

The main purpose of this study was to compare the immediate effects of Kegel exercise alone, hip muscle group contraction training at different degrees, and 5 training methods combined with training on the thickness of the TrA to explore the optimal training method for activation of that muscle.

The results of this study can be used to compare the effectiveness of methods for immediate activation of the TrA, providing an objective basis for core muscle training, related treatments, and research in clinical patients, thus providing rehabilitation guidance for patients with chronic lower back pain, rectus abdominis muscle separation, hemiplegia, and other conditions.

## 2. Methods

### 2.1. Study design and participants

From February 2024 to April 2024, 28 healthy young women were recruited from the China Rehabilitation Research Center.

The inclusion criteria: healthy women aged –20 to 25 years old; body mass index between 18.5 to 23.9 kg/m^2^; no history of urinary incontinence or other pelvic floor muscle dysfunction; no cognitive behavioral impairment; voluntarily participating in this test and signing an informed consent form.

The exclusion criteria: pregnancy; urinary tract infections and vaginal infections; neurological disorders; and hip, abdominal, and pelvic surgeries performed within the past 6 months.

This study was approved by the Medical Ethics Committee of the China Rehabilitation Research Center (No. 2024-007-1). All research subjects provided informed consent to participate in this study and signed an informed consent form. Clinical Trials registry number is ChiCTR2300074915. Information on the subjects is included in Table 1.

**Table 1:** Information on the subjects $\bar{x}\pm \;s$.

n	Age (yr)	Height (m)	Weight (kg)	BMI (kg/m^2^)
28	22.180 ± 1.541	1.643 ± 0.057	56.375 ± 6.596	20.839 ± 1.479

BMI = body mass index.

### 2.2. Experiment setup

According to the inclusion and exclusion criteria, 28 healthy young women were recruited, and all underwent Kegel exercises, mild hip adductor muscle contraction, moderate hip adductor muscle contraction, mild hip external rotation muscle contraction, and moderate hip external rotation muscle contraction trainings. The order of the 5 trainings for the same participant was randomized, and the random order was sorted using the built-in function in Excel. The resting and TrA thicknesses after each training session were measured using musculoskeletal ultrasonography. Prior to performing the Kegel exercises, participants were instructed to drink 500 mL of water and hold their urine for 1 hour. Studies have shown that this time is required to achieve optimal bladder filling.^[19]^

### 2.3. Kegel exercise

The subject lies supine with a pillow under their head, bends their hips and knees at 60°, and their feet are on the bed at the same width as their hips. The contraction of the pelvic floor muscles was controlled for 10 seconds, followed by relaxation of the muscles and holding for 10 seconds.^[20]^ The above movements were repeated 10 times, which was a set of Kegel exercises. Perform 2 sets in total, with a 20 second break between each set. The patient was instructed to “ tighten the anus.”

**Figure 1.**
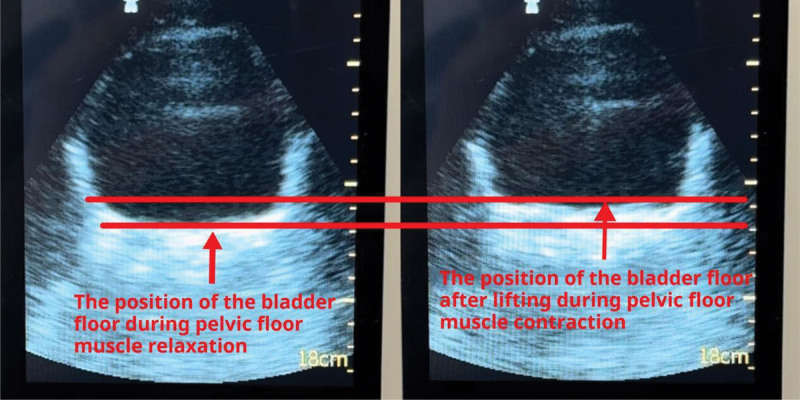
Observation of bladder floor elevation during pelvic floor muscle contraction using ultrasound.

First, ultrasound was used to observe whether the bottom of the bladder is lifted during Kegel exercises when the bladder is filled to determine whether the subject’s Kegel movements are correct (Fig. 1). The stable upward motion of the bladder bottom observed under ultrasound indicates correct pelvic floor muscle contraction, whereas the downward movement of the bladder bottom (direct downward or sudden significant downward drop during the upward motion) indicates a movement error.^[21]^ Before the training, this study ensured that the participants’ Kegel exercises were correct before starting the training.

### 2.4. Contraction degree of hip adductor muscle group

Muscle contractions were assessed using an ANIMA freehand dynamometer (ANIMA Inc., Tokyo, Japan). With Kegel movement, the subject clamped the dynamometer with maximum force and read the maximum contraction force of the hip adductor muscle group. 20% to 30% of the maximum contraction force was taken as a mild and 60% to 70% as a moderate contractions, respectively.^[8]^ Twenty mild and 20 moderate contractions were performed for each hip adductor muscle group, 10 times as a group, a total of 2 groups, with resting for 20 seconds between each group.

### 2.5. Contraction degrees of the external rotation muscles of the hip

**Figure 2.**
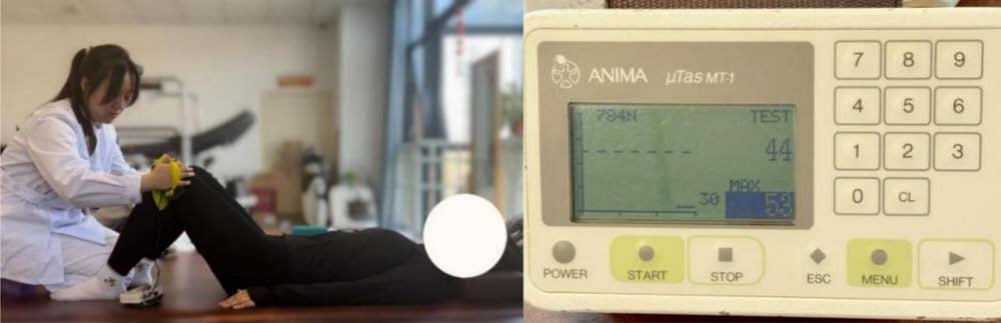
A dynamometer evaluates the degree of muscle contraction.

Muscle contractions were assessed using an ANIMA freehand dynamometer (ANIMA Inc., Tokyo, Japan). The subject’s position during Kegel exercise, subjects with the maximum force of external rotation, and the maximum contraction force of the hip external rotation muscle group were read (Fig. 2).

Twenty percent to 30% of the maximum contraction force was taken as a mild and 60% to 70% as a moderate contraction, respectively.^[8]^ Twenty mild and moderate contractions of the external rotation muscle group of the hip were performed, 10 times as a group, a total of 2 groups, resting for 20 seconds between each group.

### 2.6. Evaluation methods

TrA thickness was measured in all participants by the same researcher using the GE healthcare portable ultrasound diagnostic instrument (GE Healthcare, Hino, Japan).

**Figure 3.**
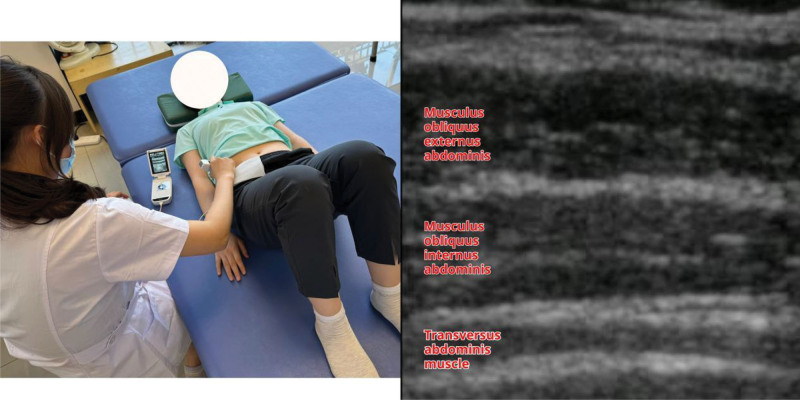
Measurement of TrA thickness. TrA = transversus abdominis.

During the test of TrA thickness, the subject took a Kegel exercise position, with intersection of the anterior axillary line and horizontal umbilical line as the center of the ultrasound probe, and gently adjusted the probe to clearly display the 3 parallel layers of the abdominal muscle structure on the screen. The anterolateral abdominal muscles were the external abdominal oblique, internal abdominal oblique, and TrA, in order from shallow to deep.^[11]^ The thickness of the TrA was measured at rest, after Kegel exercise, and after different degrees of contraction in the perihip muscle group. The images were frozen at the end of calm exhalation, and TrA thickness was measured and recorded (Fig. 3).

### 2.7. Statistical analyses

Statistical Package for Social Science (SPSS, version 26, IBM Corp., Armonk) was used for the data analysis. The measurement data conform to a normal distribution and are expressed as a mean ± standard deviation ($\bar{x}\pm s$). The comparison of TrA thickness between different training methods and duration was conducted using one-way repeated-measures analysis of variance. The significance level was set at *P* < .05.

## 3. Results

### 3.1. Comparison of TrA thickness after different training sessions

There was a significant difference (*P* < .01) in the thickness of the TrA between the different training sessions and resting states, as shown in Table 2. After 1 training session, the thickness of the TrA was greater than that at rest (*P* < .01), and after 5 training sessions, the thickness of the TrA was greater than that at rest or after 1 training session (*P* < .01), as shown in Table 3.

**Table 2:** Comparison of TrA thickness after different training sessions $\bar{x}\pm \;s$.

	Resting state	After 1 training session	After 5 training sessions	*F* value	*P* value
TrA thickness (cm)	0.231 ± 0.043	0.251 ± 0.045	0.259 ± 0.043	83.093	<.001*

TrA = transversus abdominis.

* *P* < .05.

**Table 3:** Pairwise comparison results of TrA thickness after different training sessions.

	Resting and after 1 training session	Resting and after 5 training sessions	After 1 training session and 5 training sessions
*P*	<.001*	<.001*	.002*

TrA = transversus abdominis.

* Pairwise comparison, *P* < .05.

### 3.2. Comparison of TrA thickness after 5 training methods in pairs

There was a significant difference (*P* < .01) in the thickness of the TrA immediately after different training methods, as shown in Table 4. Through pairwise comparisons, it can be concluded that the TrA thickness after moderate contraction of the hip external rotation muscle group was higher than that after mild contraction of the hip adductor muscle, moderate contraction of the hip adductor muscle, or mild contraction of the hip external rotation muscle (*P* < .01). There were no statistically significant differences between the other groups (*P* > .05), as shown in Table 5.

**Table 4:** Comparison of TrA thickness immediately after different training methods $\bar{x}\pm \;s$.

	a	b	c	d	e	f	*F* value	*P* value
TrA thickness (cm)	0.231 ± 0.043	0.252 ± 0.045	0.251 ± 0.049	0.254 ± 0.043	0.260 ± 0.043	0.256 ± 0.045	33.647	<.001*

(a) Resting. (b) Mild contraction of hip adductor muscle. (c) Moderate contraction of hip adductor muscle. (d) Mild contraction of hip external rotator muscle. (e) Moderate contraction of hip external rotator muscle. (f) Kegel exercise.

TrA = transversus abdominis.

* *P* < .05.

**Table 5:** Comparison results of TrA thickness between 5 training methods.

	b–c	b–d	b–e	b–f	c–d	c–e	c–f	d–e	d–f	e–f
*P*	.783	.532	.003*	.170	.402	.004*	.117	.006*	.476	.110

TrA = transversus abdominis.

* Pairwise comparison, *P* < .05.

## 4. Discussion

This study suggests that both the Kegel exercise and hip external rotation muscle training result in an increase of the TrA thickness, moreover, studies have shown that the increase in muscle thickness is associated with specific training movements.^[22]^ Indicating that the effects of pelvic floor muscle and hip external rotation muscle contraction on TrA activation may be consistent. From an anatomical perspective, studies have shown that beneath the peritoneum, pelvic fascia covers an upper region of the pelvic floor, covering the internal obturator, piriformis, and levator ani muscles, and continues to merge with the pubic periosteum.^[23]^ Additionally, since piriformis is one of the main muscles involved in hip joint external rotation, it may be related to pelvic floor muscle function. Tuttle et al^[24]^ found that 12 weeks of hip external rotation muscle strengthening could improve vaginal compression pressure in elderly women. Additionally, studies have shown that during pelvic floor muscle contraction, an increase in piriformis muscle thickness is observed, suggesting that pelvic floor muscle contraction may promote piriformis muscle contraction.^[25]^ This suggests that there may be a synergistic effect between the floor muscle and hip external rotation muscle group contraction, which requires further investigation.

During the process of recruiting participants, This study found that a small number of people may not be able to correctly contract the pelvic floor muscles or cannot contract continuously for 10 seconds. This is manifested by the fact that when instructing participants to contract the pelvic floor muscles, ultrasound observation showed no significant upward motion of the bladder bottom or sudden significant downward drop during the upward movement. They usually describe their self-feeling when the pelvic floor muscles contract as “unable to hold,” “uncontrollable,” “not knowing how to exert force,” etc, which makes it impossible to complete Kegel exercises. This study found that after Kegel exercises and hip external rotation muscle group contraction training, the TrA was activated immediately. Moreover, because the training movements of the hip external rotation muscle group are well understood by most people, the standard degree and standardization of the movements will be higher, and the training intensity will be easier to control and quantify. Therefore, a contraction training of the hip external rotation muscle group can be a good choice for activating the TrA, especially for people who cannot correctly complete and understand Kegel exercises.

Urinary incontinence is an objectively verifiable involuntary urinary leakage phenomenon, and it is also a disease characterized by a reduced or even lost urinary self-control due to neurological dysfunction or bladder sphincter injury.^[26]^ This study indicates that the hip external rotator muscles can immediately activate the TrA. Relevant research has shown that there is a neuroanatomical association between the TrA and pelvic floor muscles,^[27,28]^ and the 2 exhibit functional coordination (activation of the TrA promotes synchronous contraction and relaxation of the pelvic floor muscles).^[29]^ Therefore, for individuals who cannot correctly understand or perform Kegel exercises, training of the hip external rotator muscles may indirectly train the pelvic floor muscles by activating the TrA. This aspect was not explored in this study, and further research could be conducted in the future.

Instability of the lumbar spine and rectus abdominis separation are closely related to the TrA. Pre-activation and reinforcement training of the TrA can effectively promote the recovery of the abdominis separation distance.^[30]^ One possible reason for this is that the TrA is closely connected to the rectus abdominis muscle via the rectus abdominis sheath. The TrA is the deepest abdominal muscle and stabilizes the white line, ribs, and thoracolumbar fascia.^[31]^ Exercise therapy involving core-stabilizing muscles (including the TrA and multifidus muscles) is commonly used in clinical practice to treat and control chronic lower back pain.^[32]^ Core stabilizing muscles include the TrA and multifidus, among others. The TrA and multifidus have a co-contraction mechanism: when the TrA contracts, it drives the synchronous contraction of the multifidus in the back, tightly wrapping the entire lower back to maintain spinal stability. This forms a crucial protective mechanism against injury and holds significant therapeutic importance for low back pain.^[33]^ Additionally, studies have shown that muscle endurance training including the TrA can effectively reduce the incidence of low back pain.^[34]^ Contraction of the TrA can increase the tension of the thoracolumbar fascia, shrink the abdominal cavity, and increase intra-abdominal pressure. The increased intra-abdominal pressure and other related structures work together to coordinate fine motor movements between the vertebrae.^[35]^ The results of Chen et al further confirmed the importance of the TrA as a local stabilizing muscle, indicating that for hemiplegic patients, this muscle should undergo specialized training to enhance balance ability.^[36]^ Moreover, participants who had stronger trunk muscles and larger resting TrA size experienced exercise-related transient abdominal pain less.^[37]^

Choosing 1 of the 5 training methods described in this study can effectively activate the TrA in real time. However, using all 5 training methods had a better effect than a solely one method. Clinically, gymnastics training that can activate the TrA in real-time can be developed based on the results of this study. Furthermore, this study point out that moderate contraction training of the hip external rotation muscle group has the most immediate effect on activation of the TrA. Therefore, gymnastics training should include moderate movements of the hip external rotation muscle group to better activate the TrA. This may promote recovery from the chronic back pain, rectus abdominis muscle separation, hemiplegia, and other diseases in clinical practice, which requires further investigation.

Additionally, other studies have shown that the TrA thickness is significantly greater in standing rather than in the supine position.^[38]^ It is possible to consider combining the 5 training methods identified in this study that can effectively activate the TrA in real time combine with the body position. This investigation was not covered by this study and further research should be conducted in the future.

## 5. Study limitations

The limitations of this study include its small sample size and the fact that all participants were healthy young women. Additionally, ultrasonography alone was used as a single evaluation method. Furthermore, the conclusions of this study only reflect the immediate effects after training, rather than long-term impacts.This study hope these will be improved and enhanced in future studies.

## 6. Conclusions

For healthy young women, choosing 1 of 5 training methods, that is, Kegel exercise, mild and moderate contraction training of the hip adductor muscle group, and mild and moderate contraction training of the hip external rotation muscle group, can effectively activate the TrA in real-time. The combination of the 5 training methods was more effective in activating the TrA than a single training technique.

Among the 5 training methods, moderate contraction training of the hip external rotation muscle group had the best immediate activation effect on the TrA.

## Author contributions

**Conceptualization:** Yufei Gao, Miao Ye.

**Data curation:** Lili Yu.

**Formal analysis:** Yufei Gao.

**Funding acquisition:** Miao Ye.

**Investigation:** Yufei Gao.

**Methodology:** Yufei Gao.

**Resources:** Desheng Li, Miao Ye.

**Supervision:** Cong Chen, Desheng Li, Miao Ye.

**Validation:** Lili Yu, Cong Chen, Desheng Li.

**Writing – original draft:** Yufei Gao.

**Writing – review & editing:** Lili Yu, Miao Ye.
